# Do private health providers adhere to National Tuberculosis Guideline while assigning treatment outcome? Findings from a lower middle-income country

**DOI:** 10.3389/fpubh.2022.924132

**Published:** 2022-09-21

**Authors:** Victor Abiola Adepoju, Olusola Adedeji Adejumo, Oluwatoyin Elizabeth Adepoju, Marius Olusola Adeniyi, Victoria Etuk, Iheoma Nzekwe, Jude O. Inegbeboh, Ademola Adelekan, Olanrewaju Oladimeji

**Affiliations:** ^1^Department of HIV and Infectious Diseases, Jhpiego (an Affiliate of John Hopkins University), Abuja, Nigeria; ^2^Department of Community Medicine and Primary Health Care, Lagos State University Teaching Hospital, Lagos, Nigeria; ^3^Department of Adolescent Research, Adolescent Friendly Research Initiative and Care (ADOLFRIC), Ado-Ekiti, Nigeria; ^4^Department of Primary Healthcare Services, Ondo State Primary Healthcare Development Agency, Akure, Nigeria; ^5^International Research Center of Excellence (IRCE), Institute of Human Virology of Nigeria, Abuja, Nigeria; ^6^Department of HIV/AIDS, Birnin Kebbi, Kebbi State Children Emergency Fund (UNICEF), Abuja, Nigeria; ^7^Blue Gate Research Institute, Ibadan, Nigeria; ^8^Department of Public Health, Faculty of Health Sciences, Walter Sisulu University, Mthatha, South Africa

**Keywords:** tuberculosis, adherence, guideline, sputum, acid fast bacilli, treatment success rate, NTP

## Abstract

**Background:**

Treatment success rate is an important indicator to measure the performance of the National Tuberculosis Program (NTP). There are concerns about the quality of outcome data from private facilities engaged by NTP. Adherence of private providers of tuberculosis care to NTP guideline while assigning treatment outcomes to patients is rarely investigated. We aimed to determine whether Lagos private for-profit (PFP) and private not-for-profit (PNFP) facilities adhere to domestic TB guideline while assigning treatment outcome and the availability of periodic sputum acid-fast bacilli (AFB) results.

**Method:**

A retrospective review of facility treatment register and treatment cards of TB patients managed between January and December 2016 across 10 private directly observed treatment short-course (DOTS) facilities involved in the public–private mix (PPM) in Lagos, Nigeria. The study took place between January and June 2019.

**Results:**

Of the 1,566 patients, majority (60.7%) were male, >30 years (50.2%), HIV-negative (88.4%), and attended PNFP (78.5%). The reported treatment success rate (TSR) was 84.2% while the actual TSR was 53.8%. In total, 91.1, 77.6, and 70.3% of patients had sputum acid-fast bacilli (AFB) at 2/3, month 5, and month 6, respectively, while 68.6% had all the three sputum AFB in the register. Healthcare workers (HCWs) were adherent in assigning treatment outcome for 65.6% of TB patients while 34.4% of patients were assigned incorrect treatment outcomes. Most variations between reported and actual treatment outcomes were found with cured (17%) and completed (13.4%). Successful and unsuccessful outcomes were overreported by 30.4% and 4.1%, respectively. DOTS providers in private facilities with available TB guideline (OR 8.33, CI 3.56–19.49, *p* < 0.0001) and PNFP facility (OR 4.42, CI 1.91–10.3, *p* = 0.001) were more likely to adhere to National TB Guideline while assigning TB treatment outcome.

**Conclusion:**

Frontline TB providers in Lagos private hospitals struggled with assigning correct treatment outcome for TB patients based on NTBLCP guideline. Increased access to all the periodic follow-up AFB tests for TB patients on treatment and availability of National TB Guideline for referencing could potentially improve the adherence of private TB service providers while assigning TB treatment outcomes.

## Background

Nigeria is one of the 30 high-burden countries for tuberculosis (TB), tuberculosis/HIV co-infection, and multidrug-resistant tuberculosis (MDR-TB). Annually, Nigeria accounts for 9% of TB cases missed globally either because they were not diagnosed, or they were diagnosed but not reported to the National TB, Buruli Ulcer and Leprosy Control Program (NTBLCP) (1). Only 104,000 of the estimated 434,000 TB cases in Nigeria were notified in 2018 (2). In contrast, Nigeria has consistently reported treatment success rate (TSR) that is close to the 90% global target over the years (2). Successful treatment outcome is defined as the sum of patients cured and those who have completed treatment (1). The international target is to successfully treat at least 90% of new sputum smear-positive TB cases (1). In 2015, the overall TB treatment success rate in Nigeria was 87% (with a cure rate of 78% and completion rate of 8.8%) while TSR was 84% in 2016 (3–5). At the subnational level, the tuberculosis cure rate in Lagos grew from 64% in 2003 to 76% in 2014 (6). Also, 77% of a cohort of multidrug-resistant TB patients initiated in 2013 was reported to be successfully treated in 2015 compared with the 56% recorded globally (7, 8). TSR for tuberculosis provides a useful indicator to measure the quality of health services and the performance of the NTBLCP. Suboptimal TSR suggests that infectious patients may not be receiving adequate treatment. TSR has been benchmarked at 90%, and underperforming National TB Programs (NTPs) are usually placed on closer monitoring to improve the performance. Evaluation of successful treatment outcomes of new smear-positive pulmonary TB patients could also be used to determine the effectiveness of DOTS implementation.

The World Health Organization (WHO) estimated that the prevalence of MDR-TB was 4.3% among new TB cases and 25% among retreatment cases, respectively (5). Although treatment coverage is still low in Nigeria, a successful TB control program should match high treatment success rate with low figures of reported MDR-TB cases and vice versa. In addition to the poor treatment coverage, there were concerns about the accuracy of TSR figures reported by health facilities and adherence to NTBLCP guidelines while assigning those treatment outcomes. Nonadherence to guideline often manifests as incomplete documentation, underreporting and overreporting of treatment outcomes.

A study by Measure Evaluation identified nine overarching barriers to Health Management Information Systems (HMIS) in low- and middle-income countries (LMICs). These include insufficient skills in data use core competencies; poor data quality (completeness, validity, reliability, and timeliness of reporting); insufficient expertise in data synthesis and visualization; lack of systems thinking in HMIS design and development; lack of leadership and culture of data use, and, finally, poor knowledge and motivation of healthcare workers on data use (9). In Nigeria, variances between TB data reported at facility and LGA levels, the lack of data for planning, incomplete and delayed quarterly reporting, enormous missing records in health facilities, poor storage of surveillance data, and weak workforce capacity in data management have also been reported (10, 11). Custodians and managers of TB data oftentimes are not provided with the logistics needed for tracking patient lost to follow-up, conduct contact tracing, and supervise and collate data with far-reaching implications on quality and completeness of TB data (12).

A previous comparative evaluation of the performance of the public and private healthcare system in low- and middle-income countries suggested that private sector providers of TB services frequently failed to adhere to guidelines and medical standards of care leading to poor patient outcomes. The authors argued that the efficiency of the data reporting system is worse in the private sector (13). A World Health Organization (WHO) survey in Mexico showed that private practitioners managed one-third of patients who died from TB (14). Also, 85% of DR-TB patients were reported to have been previously managed for TB in the private sector (15). Despite the relatively high reported overall national TSR over the years, DR-TB case notification in Nigeria has increased from 21 in 2010 to 1,686 in 2016 and a further increase of 35% from 1,686 in 2016 to 2,286 in 2017 (2). Similarly, from 665 MDR-TB cases notified in 2013, the number of cases increased to 29,469 in 2020. Disaggregation of TSR data into the private sector component is often lacking in the annual national TB report. There is also a lack of data to confirm previous concerns that many TB patients managed in the private facilities were poorly managed and that private facilities contributed to poor TSR. This raises concerns about the reported treatment outcome data from the private sector. Analysis of adherence of staff in assigning patient outcome can help focus supervision and mentorship program to enhance the quality of care in the private sector. Therefore, we aimed to investigate adherence to the National TB Guideline in assigning treatment outcome to bacteriologically diagnosed TB patients in private facilities, Lagos, Nigeria. The specific objectives of the study include: (1) to investigate the availability of sputum AFB follow-up test needed to assign treatment outcome at various stages of TB treatment, (2) to determine the level of adherence of healthcare workers (HCWs) to NTBLCP guideline while assigning TB treatment outcome and adherence predictive factors, and (3) to highlight variations between reported and actual treatment outcomes based on standard NTBLCP case definitions for treatment outcomes in Nigeria.

## Method

### Study design

A retrospective study among all bacteriologically positive TB patients as recorded in the facility TB treatment register between January and December 2016 in Lagos, Nigeria.

### Study setting

Lagos state has a population of 24 million people and is divided into 20 Local Government Areas (LGAs) (16).

Health care in Nigeria is organized into primary, secondary, and tertiary care, and the governance of the National TB Program (embedded within the Federal Ministry of Health) is also organized in alignment with the federal system of government. The NTBLCP was established in 1989 and saddled with policy development, tertiary care, mobilization and development of human and material resource, coordination and provision of technical support to state programs. TB control activity is coordinated at the national level by the NTBLCP coordinator, at the state level by the State Tuberculosis, Buruli Ulcer and Leprosy Control Officer (STBLCO), and at the LGA level by the LGA TB, Buruli Ulcer and Leprosy supervisor (LGATBLS) assisted by the DOTS officer in the facilities (17). The health facility focal person known as the directly observed treatment short-course (DOTS) officer captures each presumptive patient in the TB presumptive register, after the preliminary test has been conducted. Once the patient is confirmed TB-positive, the details of the patient are registered in the facility TB treatment register. Records of registered TB patients are collated monthly by facility DOTS provider and sent to the LGA supervisor who will now report to the STBLCO at the end of the quarter. The STBLCO subsequently reports the state data to the NTBLCP. Similarly, TB treatment outcome data are reported in cohorts on a quarterly basis, taking cohorts of patients who initiated TB treatment in the preceding year. There are 774 LGA supervisors in the country. They all report to the state TB control officers in their respective states. The state TB officers then transmit the data to the national level (18). Also, there are six zonal arrangements at the subnational level. Each zone comprises six states. Reports from the six zones are collected and aggregated at the national level for the production of quarterly report and presentations. The NTBLCP also generates annual report based on the aggregated data from the 36 states and the Federal Capital Territory. Data validation takes place monthly or quarterly depending on the availability of funds although priority is given to public facilities and high-volume private hospitals. Electronic reporting of TB data starts at the state level when transmitting state data to the NTBLCP.

Lagos harbors 11% of the Nigerian population. Each of the Lagos LGA is supervised by an LGA TB supervisor. Healthcare service in Lagos is provided at three levels: primary, secondary, and tertiary. The Lagos State TB, Buruli Ulcer and Leprosy Control Program (LSTBLCP) was inaugurated in 2003 and expanded to engage the private sector in 2008. By the end of 2016, private facilities engaged in Lagos for tuberculosis program have increased from 8 to over 150. Facilities are often engaged under four service schemes, i.e., referral of presumptive TB only, provision of directly observed therapy short-course (DOTS) treatment only, provision of microscopy service only, and provision of both microscopy and DOTS. LSTBLCP is responsible for the training of healthcare workers and the provision of reagents, recording, and reporting tools such as the presumptive TB register, TB treatment register, and treatment cards, among others. All individuals having symptoms of TB in particular, cough of two weeks or more, are regarded as presumptive TB and documented in the presumptive TB register (17).

### Participants

A total of 10 facilities were recruited for this study. They provide a range of TB services including AFB microscopy and DOTS. Only five (50%) of the 10 centers provide AFB microscopy, and one of them also provides molecular testing services. These facilities are all private and have been engaged by TB program for at least 1 year. All the facilities recruited also provide TB/DOTS service.

**Figure 1:**
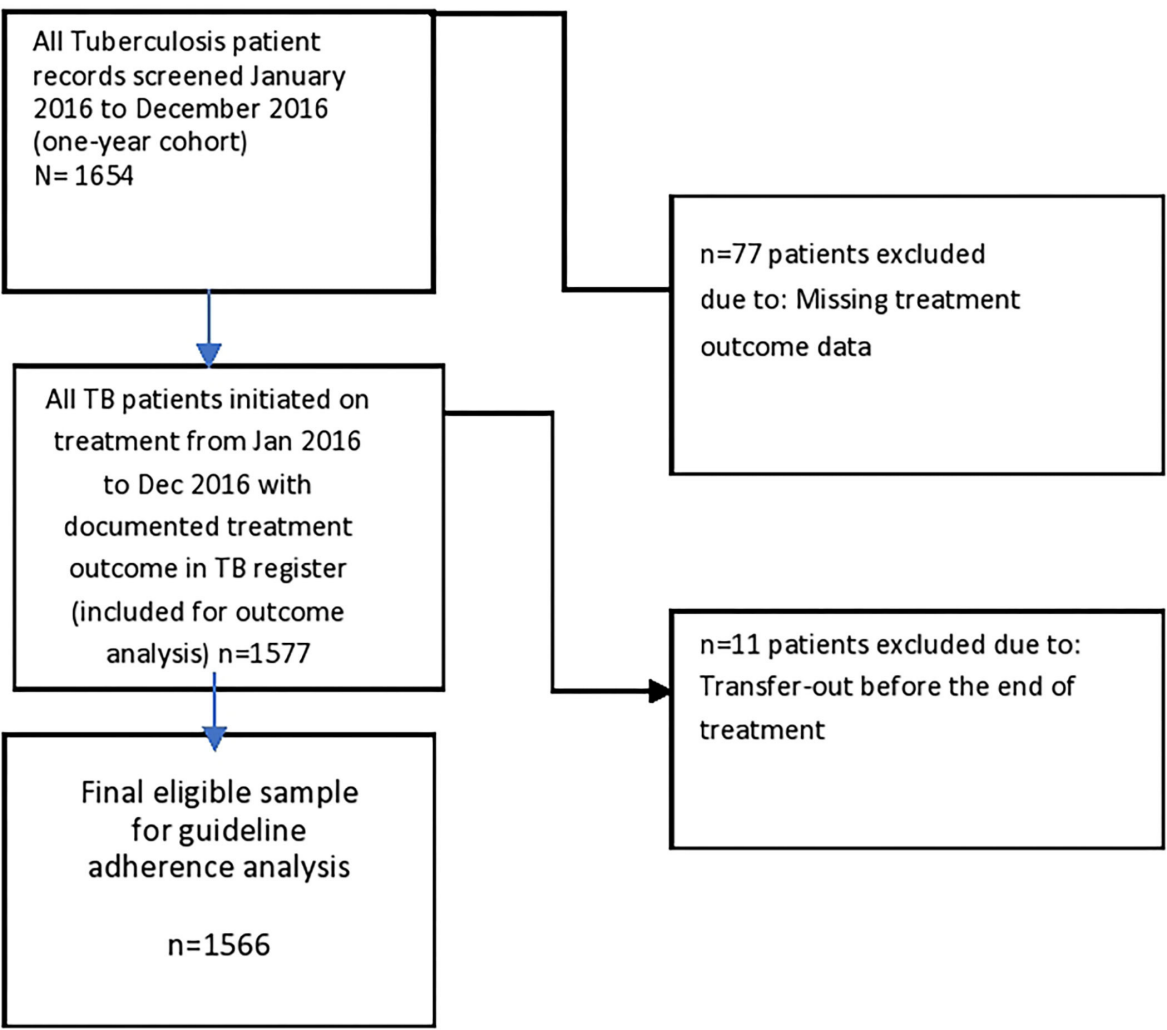
Flowchart on patient selection for analysis of adherence to National Guideline in assigning TB treatment outcome.

In Figure 1, a total of 1,654 patients (across the 10 facilities) extracted from the facility treatment register are included in the study. A total of 4.7% (77/1,654) had missing treatment outcome and were excluded from the analysis of the initial treatment outcome. Of the remaining 1,577 patients with assigned treatment outcome in the register, 11 (0.7%) were excluded from the final analysis of adherence to NTBLCP guideline since they were transferred out before the end of treatment. A total of 1,566 patients were included in the final adherence analysis.

### Bias

Where an outcome was given but AFB results were missing in the TB treatment register, a second-level check of the treatment card was conducted. In such a scenario, any missing AFB result in treatment register now found in the TB treatment card was used to update the TB treatment register accordingly.

### Study size

A sample size of all the 1,654 patients (across 10 private facilities) was extracted from the facility treatment register and included in the study.

### Sampling technique

A four-stage sampling technique (summarized below) was used to select facilities for this study.

- Stage 1: Purposive sampling technique was used in selecting the seven high TB burden LGAs in Lagos State out of the total of 20 LGAs.
- Stage 2: The TB facilities were stratified into private and public, and convenient sampling technique was used to select the private facilities. This was because of less bureaucratic processes in getting access to data from private than public facilities.
- Stage 3: Ten (10) out of 57 facilities across the seven high TB burden LGAs were selected proportionately. This implies that the facilities were selected based on the numbers of facilities in each LGA.
- Stage 4: All pulmonary tuberculosis patients were extracted from the facility treatment register.

### Data sources and measurement

Data were collected by six trained data clerks using Microsoft Excel data collection template. Information from the TB treatment register was inputted on this template. In minimizing bias in data extraction, data clerks were trained on the data collection processes. The research was also piloted in a community outside the study setting to test the practical knowledge of data collection by the trained data clerks. The objective of the study was also masked from data clerks. Daily reviews were held with data collectors to assess collected data for any missing information or double counting. No double counting was observed during the process. Prior to analysis, collected data by data clerks were triangulated with different data collection sources such as the patient treatment card and the TB treatment register. Collected data were also randomly picked by multiple observers to ensure interrater reliability. For each patient, data were collected on the date of registration, age, sex, patient category, type of facility, regimen type, date of commencement of treatment, referral setting, site of TB, means of diagnosis of TB, follow-up AFB result and grade, treatment outcome, HIV result, and cotrimoxazole preventive therapy (CPT) status. For each health facility, data were also collected on for-profit status, availability of TB guideline, provider training, refresher training, and year of training. Using these pieces of information, assigned treatment outcomes were manually linked to their test results under the result columns and interpreted against the case definition of treatment outcome assigned to determine if the assigned outcome for the patient was correct (adhere to NTP guideline) or incorrect (did not adhere to NTP guideline). Information on follow-up AFB was either collected directly from the TB treatment register or updated from TB care card if available. Adherent or non-adherent status was registered against each patient after evaluation depending on whether the assigned outcome met the case definition or otherwise.

### Definitions of terms

#### Cure

Patient who is sputum smear-negative in the last month of treatment and on at least one previous occasion (17).

#### Treatment completed

A patient who has completed treatment but who does not meet the criteria to be classified as a cure or a failure (17).

#### Treatment failure

Patient who is sputum smear-positive at 5 months or later during the treatment (17).

#### Died

Patient who died for any reason during the treatment (17).

#### Lost to follow-up

Patient whose treatment was interrupted for two consecutive months or more (17).

#### Transfer out

Patient who has been transferred to another recording and reporting unit and for whom the treatment outcome is not known (17).

#### Treatment success

It is defined as the sum of patients cured and those who have completed the treatment (17).

### Statistical method

The proportion of bacteriologically diagnosed TB patients in the facility tuberculosis treatment register that had follow-up sputum smear results available at month 2/3, month 5, and month 6 was calculated using frequency and percentages. Among those with available smear follow-up results, the proportions with smear conversion were also estimated and used to determine actual treatment outcomes. Among bacteriologically confirmed TB patients with end-of-treatment (month 6) smear result, the proportion of each outcome (cure, failure, treatment completed) as derived from the TB treatment register was validated for adherence against case definitions provided by NTBLCP guideline and marked as adhered (correct outcome) or non-adherent (incorrect treatment outcome). Descriptive statistics such as frequency and percentages were used to present adherence level and availability of sputum AFB follow-up result. In addition, predictors of healthcare workers' adherence to TB guideline were also determined at univariate and multivariate levels using Statistical Program for Social Sciences, version 17, at a level of significance *p* < 0.001 and confidence interval of 95%.

### Ethical approval

As data for this study were retrieved from routinely collected surveillance register of the LSTBLCP, no ethical clearance was required. Permission was received from the Lagos State Ministry of Health. Patient information was deidentified before the analysis for confidentiality.

## Results

### Descriptive data

In Table 1, of the 1,577 eligible records, majority (60.7%) of the participants were male, >30 years (50.2%), HIV-negative (88.4%), and attended private not-for-profit (78.5%). In total, 0.7% of the patients were transferred out.

**Table 1:** Demographic characteristics and reported treatment outcomes for TB patients who initiated treatment across 10 private hospitals in Lagos State between January–December 2016 (*n* = 1,577).

**Variable**	**Number**	**Percentage**
**Patient age**
0–30 years	785	49.8
>30 years	992	50.2
**Sex**
Male	957	60.7
Female	620	39.3
**Type of hospital**
Private for profit	339	21.5
Private not for profit	1,238	78.5
**TB service scheme**
3a	8	80
3b	2	20
**Provider training (year)**		
≤ 2012	7	70
>2012	3	30
**TB refresher training**
Yes	2	20
No	8	80
**Guideline availability**
Yes	4	40
No	6	60
**HIV status**
Positive	100	6.3
Negative	1,394	88.4
Unknown	83	5.3
**Treatment outcome**
Cured	1,064	67.5
Completed	255	16.1
LTFU	186	11.8
Died	47	3.0
Treatment failure	14	0.9
Transfer out	11	0.7
**Treatment success rate (TSR)**
Successful	1,319	83.6
Not successful	258	16.4

### Outcome data

In Table 2, 91.1, 77.6, and 70.3% of patients were respectively offered sputum smear microscopy at months 2/3, month 5, and month 6 in that decreasing order. In total, 68.6% of patients in our study had all the three sputum follow-up results.

**Table 2:** Analysis of availability of Acid-Fast Bacilli (AFB) Sputum Follow up result at months 2/3, 5 and 6 in Tuberculosis Treatment Register (*n* = 1,566).

**Follow-up month**	**Number (*N*)**	**Percentage %**
**Month 2/3**
0	1,332	85.6
Scanty	10	0.6
1+	78	5.0
Not done	146	9.0
Done	1,420	91.0
**Month 5**
0	1,198	76.4
Scanty	2	0.1
1+	18	1.1
Not done	348	22.2
Done	1,218	77.6
**Month 6**
0	1,096	70.0
Scanty	1	0.06
1+	5	0.3
Not done	464	29.8
Done	1,102	70.3
**Months 2/3,5&6**	1,074	68.6

Table 3 highlights the level of adherence to NTBLCP guideline while assigning treatment outcome to TB patients. Overall, HCWs were adherent in assigning treatment outcome for 65.6% of TB patients while 34.4% of patients were assigned incorrect treatment outcome. Nonadherence was highest when assigning “treatment completion,” 82% (209/255), followed by treatment failure (57.1%) and LTFU 29.6% (55/186) but least for death outcome 0% (0/14) and cure, 25.1% (267/1,064). Most variations between reported and actual treatment outcomes were found with cured (17%) and completed (13.34%) outcomes. Successful and unsuccessful outcomes were overreported by a difference of 30.4 and 4.1%, respectively.

**Table 3:** Analysis of level of adherence to National TB Guideline in assigning various TB treatment outcomes (*n* = 1,566).

**Indicator**	**Not adherent (incorrect)%**	**Adherent (correct)%**	**Reported treatment outcome (%)**	**Actual treatment outcome (%)**	**% Diff (actual-reported)**
Cured	267 (25.1)	797 (74.9)	1,064 (67.9)	797 (50.9)	17.0
Completed	209 (82.0)	46 (18.0)	255 (16.3)	46 (2.94)	13.36
Died	0 (0.0)	47 (100)	47 (3.0)	47 (3.0)	0.0
Failure	8 (57.1)	6 (42.9)	14 (0.9)	6 (0.38)	0.52
LTFU	55 (29.6)	131 (70.4)	186 (11.9)	131 (8.4)	3.5
Successful outcome	476 (36.1)	843 (63.9)	1,319 (84.2)	843 (53.8)	30.4
Unsuccessful outcome	63 (25.5)	184 (74.5)	247 (15.8)	184 (11.7)	4.1
Overall adherence	539 (34.4)	1,027 (65.6)			

% Adherence, # of correct outcome/# same category of outcome; % Non-adherent: # of incorrect outcome/# same category of outcome; % Reported, Treatment outcome Total # of specific outcome category/Total # of patients (1,577); % Actual treatment outcome, # of correct outcome out of same reported outcome category/Total # of patients (1,577); %Diff in outcome, Diff in Reported and Actual patient/Total # of patients (1,577).

In Table 4, multivariate analysis shows that DOTS providers in private facilities with available TB guideline (OR 8.33, CI 3.56–19.49, *p* < 0.0001), PNFP facility (OR 4.42, CI 1.91–10.3, *p* = 0.001) were more likely to adhere to National TB Guideline while assigning TB treatment outcome.

**Table 4:** Facility and DOTS provider correlates of adherence to National TB Guideline.

**Variable**	**Non-adherent**	**Adherent**	**cOR, 95% CI**	**aOR, 95% CI**	***p*-Value**
**Facility**
PFP	98 (28.9)	241 (71.4)		Ref	
PNFP	452 (36.5)	786 (23.5)	0.71 (0.5–0.9)	4.42 (1.91–10.3)	0.001
**Refresher training**
No	26 (32.5)	30 (41.7)	1.09 (1.07–1.11)	–	1.000
Yes	54 (67.5)	42 (58.3)			
**TB guideline**
No	9 (11.3)	4 (5.6)			
Yes	71 (88.8)	68 (94.4)	0.99 (0.8–1.3)	8.33 (3.56–19.49)	< 0.0001
**TB service scheme**
3a	27 (33.8)	24 (34.7)	0.53 (0.3–0.9)	1.62 (0.82–3.19)	0.162
3b	53 (66.3)	47 (65.3)			

cOR, crude odd ratio; aOR, adjusted odd ratio; 3a, TB treatment services only; 3b, TB diagnosis and treatment.

## Discussion

The study aimed to investigate whether private hospital TB service providers assigned correct treatment outcome to tuberculosis patient based on case definitions in NTBLCP guidelines. The study highlighted the availability of periodic sputum AFB results in treatment records as well as variations in reported and actual treatment outcome and predictive factors. In total, 4.7% of patient entries in this study had missing treatment outcome. This is lower than findings from Lagos and Kenya studies where 53.8 and 30% of records had missing treatment outcomes, respectively (19, 20). Healthcare workers might not document treatment outcome if they were not able to retrieve complete sputum AFB results from the laboratory. It is also possible that some of the laboratory results got missing before they were documented. The above findings underline the need to strengthen interventions that improve patient access to follow-up test and results, particularly month 6 result in the absence of which “cured” outcome cannot be declared.

Only 65.6% of TB patients received correct treatment outcome based on current domestic NTBLCP guideline for frontline healthcare workers. This contrasts with the Lagos study where reported treatment success was associated with full adherence (19). However, the study only mentioned that 53.8% of treatment outcome records were missing but failed to describe how adherence to treatment outcome for patients managed in the private sector was specifically measured rather than specified (19). All the 34 DOTS sites recruited in the study had microscopy services which may indicate better access; unlike in our study, where only half of the facilities had AFB services.

In the current study, reported lost to follow-up (LTFU) was 11.9%, but the validated figure was 8.4% giving a difference of 3.5%. The figure is lower than findings from Nepal in which 16.8% of patients assigned LTFU were found to have completed treatment during community tracking and validation by NTP (21, 22). It is possible that providers erroneously assigned LTFU for TB patients yet to fulfill LTFU case definition or were unable to update TB registers for TB patients who later presented at health facilities after the initial missed appointments. In Botswana, for instance, 39% of sputum smears were declared as not done instead of a lower figure of 16%. This gap was due to the failure of documentation by HCWs. This finding highlights why emphasis should be placed on knowledge of case definitions of TB treatment outcomes by private providers during supervisory visits, the need for home tracking, and prompt recording (23).

In this study, the percentage difference in the reported (documentation in register) and validated/actual (NTBLCP case definitions) treatment completed and cure rates were 13.4% and 17%, respectively. The investigators noted that as against the reported cure rate of 67.9%, the validated figure was 50.9%, while the completion rate was validated as 2.94% as against the reported completion rate of 16.3%. Overall, treatment success in this study was overreported by a difference of 30.4%. This figure is higher than findings from South African study where TSR was overreported by 12% (24) but lower than findings from KwaZulu natal study where only 34.8% of the reported cure rate was validated (25). It was obvious in our study that HCWs poorly understood how ‘treatment completed' differed from ‘cured'. Many patients who had negative month 6 AFB result with at least one previous negative AFB result were still labeled as completed in the treatment records, while several others without month 6 follow-up test were assigned cured. This highlights the need to structure supervisory and training program to specific needs of private providers with the view to bridging gaps in knowledge and health systems performance. Overreporting of cure rate can give a false impression of TB control. If patients are managed according to their smear results and their follow-up smear is inaccurately interpreted, healthcare workers may be inappropriately and unknowingly treating drug-resistant TB patients with first-line anti-TB drugs.

We also found that PNFPs (compared to PFPs) were four times more likely to adhere to national guideline while assigning treatment outcome while DOTS providers in facilities with National TB Guidelines (compared to those with no guideline) were eight times more likely to adhere to guideline when assigning TB treatment outcomes. These findings have implications for policy and practices. A study from Northern Nigeria found that the poor quality of TB services in the private sector is impacting TB treatment outcome (26). These differential findings in the documentation among private providers by profit status could mean that PFPs fear that adhering to the TB guidelines would reduce the monetary profit they will make from treating TB patients. These findings are pertinent because they are critical to the use of the guidelines by the practitioners and subsequent good outcomes for patients treated in private health facilities.

Guidelines traditionally serve as reference guide and means of refresher for providers across different knowledge areas, for example, clinical management, monitoring and evaluation, documentation and reporting. Prioritization of public sector facility while disseminating such guidelines with private sector left out is not uncommon. Studies have shown that 60% of guideline available at the central level were not available at service delivery point and over 50% of nurses did not document nursing care provided (27, 28). Making these guidelines available at lower level of care could help providers to reference and clarify technical issues toward improving on documentation and reporting practices. Such guidelines need to be precise, less bulky for busy private sector providers who oftentimes backstop for multiple disease areas including TB services. There is a need for NTBLCP to intensify structured training and supervision/mentorship visits targeted at private facilities with emphasis on case definitions of treatment outcomes. The approach could change the narrative in the private sector where data issues may be a big challenge due to the limited human resources needed to provide quality TB services. Innovative, real-time digitization of reportable case-based TB indicators could help to reduce the volume of paper-based documentation, improve the quality and fidelity of reported TB data, and help in real-time decision-making toward the realization of TB control and elimination goals (29).

### Limitation

One of the limitations of this study is that follow-up results were not traced to the laboratory since there was no digital linkage of the laboratory with facility TB surveillance system. However, the objective was not to validate results with laboratory records but to check for the adherence of HCWs and the correctness of the assigned treatment outcome based on the case definitions of the NTBLCP guidelines. We did a second-level check for missing AFB results in the TB treatment card which reduced the possibility of underestimation of treatment outcome. In rare instances, it was possible that treatment outcomes of some patients were validated during data collation by the LGA TB supervisors before reporting to NTBLCP, although we expect such cases to have been updated in the facility TB register or patient treatment card. The study also reviewed TB surveillance data in 2017. Therefore, it will be relevant if follow-up study is done to evaluate any changes or improvement between 2017 and till date. This study is a retrospective review of facility TB register and treatment card and did not consider other factors that could have influenced the quality of TB data management.

## Conclusion

A huge percentage of patients managed in private facilities in a lower middle-income country were not assigned the correct treatment outcome based on the NTBLCP guideline. Only 68% of patients had all the three periodic sputum follow-up results needed to assign correct treatment outcomes. The huge proportion of TB patients with missing end-of-treatment, month 6 follow-up results is of great concern. The result was a huge discrepancy between the reported and actual cure and completion rate data. Giving the importance of sputum AFB results in the management of TB patients and assigning the correct treatment outcome, measures should be put in place to improve patient access to laboratory follow-up tests where TB drugs were collected. There is a need to establish systems for complete logging and tracking of all sputum samples received in the laboratory, timely retrieval, and accurate recording of results at the facility level. Linking laboratory directly with the NTBLCP surveillance system is recommended since it has the potential to minimize missing data and discrepancies and more accurately influence the quality of TB program outcome data. National TB Programs in low- and middle-income countries (LMICs) need to invest more on regular supervision, training and retraining of staff, provision of updated guidelines, and exchange mentorship from highly adherent private and public facilities to poorly adherent private facilities. In addition, we call for a business model that links the quality of data reported by the private facility providers with annual government accreditation programs. Future research should investigate the impact of removal of fees for laboratory follow-up test through health insurance programs and the impact that digitalization of TB Health Information Management Systems will have on documentation practices and the integrity of the TB treatment outcome data in the private sector.

## Data availability statement

The original contributions presented in the study are included in the article/supplementary material, further inquiries can be directed to the corresponding author.

## Author contributions

VA: conceptualization, formal analysis, methodology, validation, and writing-original draft. OAA, MA, and VA: visualization. OAA, MA, AA, and VA: writing-review and editing. OEA and IN: writing methodology. MA: writing result. VE and VO: rewriting introduction and project administration. VA and AA: overall project administration and supervision. IN, OAA, and VA: data analysis. JOI: wrote the introduction and part of the discussion. OO: wrote part of the discussion, revised the manuscript based on feedback from the reviewers. All authors contributed to the article and approved the submitted version.

## Conflict of interest

The authors declare that the research was conducted in the absence of any commercial or financial relationships that could be construed as a potential conflict of interest.

## Publisher's note

All claims expressed in this article are solely those of the authors and do not necessarily represent those of their affiliated organizations, or those of the publisher, the editors and the reviewers. Any product that may be evaluated in this article, or claim that may be made by its manufacturer, is not guaranteed or endorsed by the publisher.

## Abbreviations

AFB: acid-fast bacilli
CQI: continuous quality improvement
CPT: cotrimoxazole therapy
DOTS, directly observed therapy: short-course
DS-TB-drug: susceptible tuberculosis
ETR: electronic tuberculosis reporting
HCWs: healthcare workers
LGATBLS, Local Government Area Tuberculosis: Buruli Ulcer and Leprosy Control Supervisor
LGA: Local Government Area
LTFU: lost to follow-up
LMICs: low- and middle-income countries
LSTBLCP: Lagos State Tuberculosis and Leprosy Control Program
STBLCO, State TB: Buruli Ulcer and Leprosy Control Officer
MDR-TB: multidrug-resistant tuberculosis
NTP: National Tuberculosis Program
NTBLCP, National Tuberculosis: Buruli Ulcer and Leprosy Control Program
PPM: public–private mix
TSR: treatment success rate
TB: tuberculosis
WHO: World Health Organization.
